# Supplementary material for: Leveraging artificial intelligence to identify the psychological factors associated with conspiracy theory beliefs online
Source: Nat Commun. 2024 Aug 29;15:7497. doi: 10.1038/s41467-024-51740-9 (PMC11362279; doi:10.1038/s41467-024-51740-9)
Supplement: Supplementary file 1 — Supplementary Information [file 41467_2024_51740_MOESM1_ESM.pdf]

Supplementary Information

Leveraging Artificial Intelligence to Identify the Psychological Factors Associated With Conspiracy Theory Beliefs Online

Jonas R. Kunst[1*], Aleksander B. Gundersen[1#], Izabela Krysińska[2#], Jan Piasecki[3], Tomi Wójtowicz[2], Rafal Rygula[4], Sander van der Linden[5] & Mikołaj Morzy[2]

Supplementary Information Contents

Figure S1 | Cumulative Capture of Positives Based on Human Ratings (Majority Vote) Across Estimated Similarity Scores. 4

Dispersion Test for Main Models Present in the Manuscript 5

Supplementary Note S1. Testing for interaction between perceived ability to detect misinformation and self-reported belief in false information 6

Supplementary Note S2. Testing for curvilinear influence of perceived ability to detect misinformation 6

Supplementary Note S3. Analysis of subset of Twitter (currently X) engagements that were topically related to conspiracy theories 6

Table S1 | Conspiracy Theory 1: Standardized multi-level regression analysis predicting the likelihood that Twitter (currently X) engagements supported the conspiracy belief that the virus and response to it is aimed to create economic instability and benefit large corporations, calculated on Twitter (currently X) engagements with sim > 0.25 ($N_{Participants}$ = 2,113, $N_{Engagements}$ = 462,452). 7

Table S2 | Conspiracy Theory 2: Standardized multi-level regression analysis predicting the likelihood that Twitter (currently X) engagements supported the conspiracy belief that the public is being intentionally misled about the true nature of the virus and prevention, calculated on Twitter (currently X) engagements with sim > 0.25 ($N_{Participants}$ = 1,993, $N_{Engagements}$ = 326,380). 8

Table S3 | Conspiracy Theory 3: Standardized multi-level regression analysis predicting the likelihood that Twitter (currently X) engagements supported the conspiracy belief that the virus is human-made and a bioweapon, calculated on Twitter (currently X) engagements with sim > 0.25 ($N_{Participants}$ = 2,139, $N_{Engagements}$ = 369,568). 9

Table S4 | Conspiracy Theory 4: Standardized multi-level regression analysis predicting the likelihood that Twitter (currently X) engagements supported the conspiracy belief that governments and politicians are intentionally spreading false information, calculated on Twitter (currently X) engagements with sim > 0.25 ($N_{Participants}$ = 2,119, $N_{Engagements}$ = 565,274). 10

Table S5 | Conspiracy Theory 5: Standardized multi-level regression analysis predicting the likelihood that Twitter (currently X) engagements supported the conspiracy belief that China intentionally spread the virus to hurt other countries, calculated on Twitter (currently X) engagements with sim > 0.25 ($N_{Participants}$ = 2,015, $N_{Engagements}$ = 291,514). 11

Table S6 | Conspiracy Theory 6: Standardized multi-level regression analysis predicting the likelihood that Twitter (currently X) engagements supported the conspiracy belief that the vaccines are unsafe or a means of population control, calculated on Twitter (currently X) engagements with sim > 0.25 ($N_{Participants}$ = 2,088 , $N_{Engagements}$ = 381,835). 12

Table S7 | Conspiracy Theory 1: Association between predictors and likelihood that Twitter (currently X) engagement supported conspiracy theory by engagement type (Separate models were estimated for each type). 13

Table S8 | Conspiracy Theory 2: Association between predictors and likelihood that Twitter (currently X) engagement supported conspiracy theory by engagement type (Separate models were estimated for each type). 14

Table S9 | Conspiracy Theory 3: Association between predictors and likelihood that Twitter (currently X) engagement supported conspiracy theory by engagement type (Separate models were estimated for each type). 15

Table S10 | Conspiracy Theory 4: Association between predictors and likelihood that Twitter (currently X) engagement supported conspiracy theory by engagement type (Separate models were estimated for each type)....16

Table S11 | Conspiracy Theory 5: Association between predictors and likelihood that Twitter (currently X) engagement supported conspiracy theory by engagement type (Separate models were estimated for each type)....17

Table S12 | Conspiracy Theory 6: Association between predictors and likelihood that Twitter (currently X) engagement supported conspiracy theory by engagement type (Separate models were estimated for each type)....18

Figure S2 | Correlations Between Self-Report Measures....19

Table S13 | Two-tailed P-values for correlations shown in Figure S2. 20

Table S14 | N for correlation tests shown in Figure S2. 20

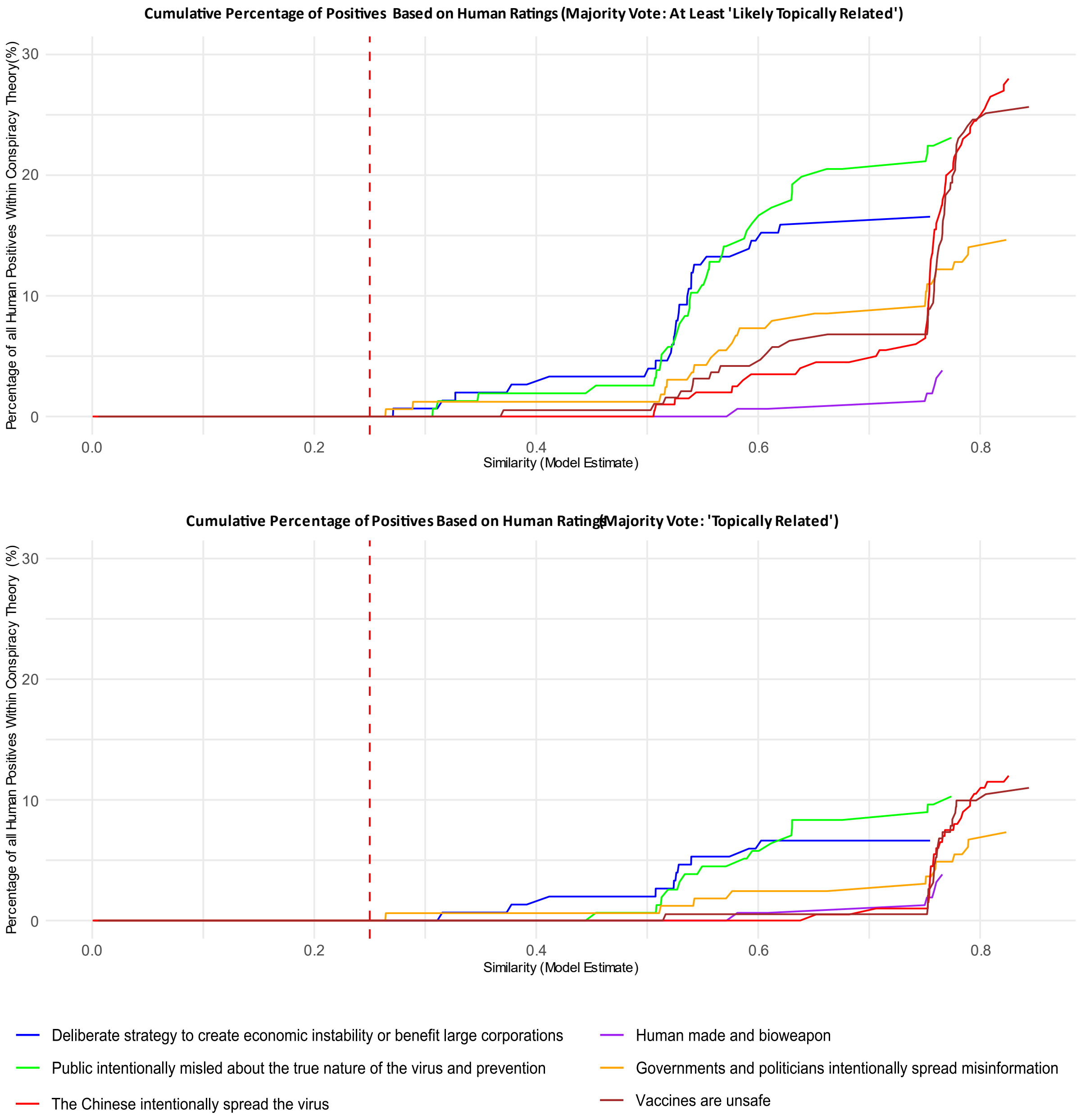

Figure S1 | Cumulative Capture of Positives Based on Human Ratings (Majority Vote) Across Estimated Similarity Scores. For the upper graph more lenient, the majority of raters had to rate a Twitter (currently X) engagement as “likely topically related” or as “topically related” to the respective conspiracy theory. For the lower more conservative graph, the majority of raters had to rate an Twitter (currently X) engagement as “topically related” to the respective conspiracy theory. The dashed red line indicates the 0.25 similarity cutoff. The different colors used are explained in the color legend below the graph. N = 1019.

Dispersion Test for Main Models Present in the Manuscript

Model 1

dispersion = 0.88565, p-value = 0.888

Model 2

dispersion = 1.1095, p-value = 0.472

Model 3

dispersion = 0.49605, $P = 0.032$

Model 4

dispersion = 1.1132, $P = 0.488$

Model 5

dispersion = 0.02427, $P < 0.001$

Model 6

dispersion = 0.58576, $P = 0.320$

Supplementary Note S1. Testing for interaction between perceived ability to detect misinformation and self-reported belief in false information

Considering the mixed findings in prior research regarding the role of the perceived ability to spot misinformation, we explored its interaction with belief in false information. It could be hypothesized that this perceived ability might predominantly bolster conspiracy theory support among those already inclined to believe in misinformation. Nonetheless, our analysis revealed that this interaction was not statistically significant when added to any of the models, βs = 0.01–0.13, all $Ps_{Holm} = 1.000$.

Supplementary Note S2. Testing for curvilinear influence of perceived ability to detect misinformation

To determine if overconfidence, rather than the perceived ability itself, drives the observed association with conspiracy theory support, we examined the possibility of a curvilinear relationship. These models were tested only controlling for participants followers However, our analysis revealed that this quadratic association was not statistically significant, βs = -0.02 – -0.16, all $Ps_{Holm} > 0.460$.

Supplementary Note S3. Analysis of subset of Twitter (currently X) engagements that were topically related to conspiracy theories

Validation results indicated that tweets with very low topical relevance to the respectively examined conspiracy theory (i.e., possessing similarity scores below 0.25, as detailed in the *Methods* section) were not expected to yield any true positives. Consequently, engagements deemed entirely unrelated to a particular conspiracy theory, based on this statistical machine-learning criterion, were classified as negative instances (i.e., assigned a score of 0). This approach allowed for the utilization of the entire sample while adhering to the resource limitations of this study, given that classifying millions of engagements falling below this threshold was deemed impractical due to their very low likelihood of containing relevant instances, as they did not pertain topically to the conspiracy theory under investigation. Nevertheless, to ensure a comprehensive analysis, the models were re-estimated for each conspiracy theory exclusively on data subsets with similarity scores above 0.25 in relation to the respective theory. It is crucial to acknowledge that this methodological adjustment alters the composition of the sample significantly: These analyses are based on individuals whose interactions to more of an extent are related to conspiracy theories and disregards approximately 20% of the total participant sample uninclined towards conspiracy theory involvement and about 90% of all Twitter (currently X) engagements. This part of the overall sample may be critical to statistically identify risk and resilience factors in a general population sample. Nevertheless, for the sake of transparency, we report results based on a subset with similarity scores > 0.25 in Tables S1 through S6 in the *Supplementary Information* and briefly summarize them here.

Results revealed that the observed patterns for the second, fifth, and sixth conspiracy theories remained the same. However, for the first and fourth theories, the previously identified curvilinear relationships lost statistical significance. Additionally, in the case of the fourth conspiracy theory, the correlation with the perceived capacity to discern misinformation also became statistically non-significant. Conversely, engagements from individuals identifying as independents were found to be more supportive of the fourth conspiracy theory, while engagements from individuals identifying as Republicans displayed a higher likelihood of endorsing the third conspiracy theory.

Table S1 | Conspiracy Theory 1: Standardized multi-level regression analysis predicting the likelihood that Twitter (currently X) engagements supported the conspiracy belief that the virus and response to it is aimed to create economic instability and benefit large corporations, calculated on Twitter (currently X) engagements with sim > 0.25 ($N_{Participants}$ = 2,113, $N_{Engagements}$ = 462,452).

		95% CI			95% CI		
Variable	β	lower	upper	*OR*	lower	upper	P_{Holm}
(Intercept)	-6.40	-6.70	-6.09	0.00	0.00	0.00	<0.001
Followers on Twitter (currently X)	-0.22	-0.68	0.25	0.81	0.50	1.28	1.000
Following on Twitter (currently X)	0.28	-0.23	0.78	1.32	0.79	2.19	1.000
Age	0.20	0.06	0.33	1.22	1.06	1.39	0.043
Education	0.10	-0.02	0.22	1.11	0.98	1.25	0.798
Gender[1]: Woman	-0.03	-0.28	0.21	0.97	0.76	1.24	1.000
Gender[1]: Other	0.09	-0.57	0.75	1.09	0.56	2.11	1.000
Political Orientation (linear)	-0.19	-0.37	0.00	0.83	0.69	1.00	0.430
Political Orientation (quadratic)	0.07	-0.05	0.20	1.07	0.95	1.22	1.000
Political Party[3]: Republican	-0.24	-0.73	0.25	0.79	0.48	1.29	1.000
Political Party[3]: Independent	0.06	-0.24	0.37	1.07	0.78	1.45	1.000
Political Party[3]: Other	0.13	-0.35	0.60	1.13	0.71	1.82	1.000
Belief in False Information	0.10	-0.05	0.25	1.10	0.95	1.28	1.000
Disbelief in True Information	-0.04	-0.18	0.09	0.96	0.84	1.09	1.000
Conspiracy Mentality	0.08	-0.08	0.23	1.08	0.93	1.26	1.000
Narcissism	-0.07	-0.19	0.06	0.93	0.83	1.06	1.000
Denialism	0.06	-0.09	0.22	1.07	0.91	1.24	1.000
Need for Chaos	0.02	-0.13	0.16	1.02	0.88	1.18	1.000
Belief in Information Reliability	-0.01	-0.14	0.11	0.99	0.87	1.12	1.000
Importance of Verifying Information	0.00	-0.13	0.13	1.00	0.88	1.14	1.000
Perceived Ability to Recognize Misinformation	0.06	-0.07	0.18	1.06	0.93	1.20	1.000
Adjusted ICC	0.229						
Unadjusted ICC	0.223						
Conditional R^2	0.249						
Marginal R^2	0.026						

A generalized linear mixed model was estimated. β = Standardized regression coefficient. CI = Confidence Intervals. OR = Odds ratios based on standardized scores. P_{Holm} = One-tailed P-test, Holm-corrected for multiple comparisons. [1]Reference group = men. [2]Higher values mean a more right-leaning political orientation. [3]Reference group = Democrats. ICC = Intraclass correlation coefficients. R^2 = explained variance.

Table S2 | Conspiracy Theory 2: Standardized multi-level regression analysis predicting the likelihood that Twitter (currently X) engagements supported the conspiracy belief that the public is being intentionally misled about the true nature of the virus and prevention, calculated on Twitter (currently X) engagements with sim > 0.25 ($N_{Participants}$ = 1,993, $N_{Engagements}$ = 326,380).

		95% CI			95% CI		
Variable	β	lower	upper	*OR*	lower	upper	P_{Holm}
(Intercept)	-3.98	-4.14	-3.82	0.02	0.02	0.02	<0.001
Followers on Twitter (currently X)	0.01	-0.28	0.31	1.01	0.75	1.37	1.000
Following on Twitter (currently X)	-0.07	-0.37	0.24	0.94	0.69	1.27	1.000
Age	0.33	0.25	0.41	1.39	1.29	1.50	<0.001
Education	-0.05	-0.11	0.02	0.95	0.89	1.02	0.854
Gender[1]: Woman	-0.17	-0.30	-0.03	0.85	0.74	0.97	0.130
Gender[1]: Other	-0.36	-0.80	0.08	0.70	0.45	1.09	0.668
Political Orientation (linear)	0.16	0.06	0.26	1.18	1.06	1.30	0.013
Political Orientation (quadratic)	0.03	-0.04	0.09	1.03	0.96	1.10	1.000
Political Party[3]: Republican	0.21	-0.04	0.46	1.23	0.96	1.58	0.633
Political Party[3]: Independent	0.16	-0.02	0.34	1.17	0.98	1.40	0.630
Political Party[3]: Other	-0.12	-0.42	0.18	0.89	0.66	1.20	1.000
Belief in False Information	0.18	0.10	0.26	1.20	1.11	1.30	<0.001
Disbelief in True Information	0.06	-0.02	0.13	1.06	0.98	1.14	0.746
Conspiracy Mentality	0.05	-0.04	0.14	1.05	0.96	1.15	1.000
Narcissism	-0.06	-0.13	0.01	0.94	0.88	1.01	0.630
Denialism	0.13	0.04	0.22	1.14	1.04	1.25	0.029
Need for Chaos	-0.05	-0.13	0.03	0.95	0.88	1.03	0.954
Belief in Information Reliability	0.02	-0.05	0.09	1.02	0.95	1.09	1.000
Importance of Verifying Information	0.04	-0.04	0.11	1.04	0.96	1.12	1.000
Perceived Ability to Recognize Misinformation	0.03	-0.04	0.10	1.03	0.96	1.11	1.000
Adjusted ICC	0.15						
Unadjusted ICC	0.14						
Conditional R^2	0.23						
Marginal R^2	0.10						

A generalized linear mixed model was estimated. β = Standardized regression coefficient. CI = Confidence Intervals. OR = Odds ratios based on standardized scores. P_{Holm} = One-tailed P-test, Holm-corrected for multiple comparisons. [1]Reference group = men. [2]Higher values mean a more right-leaning political orientation. [3]Reference group = Democrats. ICC = Intraclass correlation coefficients. R^2 = explained variance.

Table S3 | Conspiracy Theory 3: Standardized multi-level regression analysis predicting the likelihood that Twitter (currently X) engagements supported the conspiracy belief that the virus is human-made and a bioweapon, calculated on Twitter (currently X) engagements with sim > 0.25 ($N_{Participants}$ = 2,139, $N_{Engagements}$ = 369,568).

		95% CI			95% CI		
Variable	β	lower	upper	*OR*	lower	upper	P_{Holm}
(Intercept)	-7.48	-7.96	-7.00	0.00	0.00	0.00	<0.001
Followers on Twitter (currently X)	-0.51	-1.29	0.28	0.60	0.28	1.32	0.914
Following on Twitter (currently X)	0.37	-0.34	1.09	1.45	0.71	2.98	1.000
Age	0.30	0.11	0.49	1.35	1.11	1.63	0.019
Education	0.00	-0.17	0.17	1.00	0.85	1.19	1.000
Gender[1]: Woman	-0.58	-0.92	-0.24	0.56	0.40	0.79	0.008
Gender[1]: Other	-0.71	-2.10	0.69	0.49	0.12	1.99	1.000
Political Orientation (linear)	0.54	0.28	0.81	1.72	1.32	2.24	0.001
Political Orientation (quadratic)	-0.11	-0.28	0.06	0.89	0.75	1.06	0.914
Political Party[3]: Republican	0.63	0.00	1.25	1.87	1.00	3.50	0.321
Political Party[3]: Independent	0.82	0.33	1.32	2.28	1.38	3.75	0.011
Political Party[3]: Other	0.34	-0.45	1.13	1.40	0.64	3.08	1.000
Belief in False Information	0.23	0.04	0.42	1.26	1.04	1.53	0.127
Disbelief in True Information	0.14	-0.04	0.32	1.15	0.97	1.38	0.698
Conspiracy Mentality	0.07	-0.16	0.29	1.07	0.85	1.33	1.000
Narcissism	-0.21	-0.38	-0.03	0.81	0.68	0.97	0.145
Denialism	0.31	0.08	0.53	1.36	1.08	1.71	0.066
Need for Chaos	-0.15	-0.36	0.06	0.86	0.70	1.06	0.890
Belief in Information Reliability	-0.12	-0.29	0.06	0.89	0.75	1.06	0.914
Importance of Verifying Information	-0.05	-0.22	0.13	0.95	0.80	1.14	1.000
Perceived Ability to Recognize Misinformation	0.08	-0.09	0.25	1.09	0.91	1.29	1.000
Adjusted ICC	0.304						
Unadjusted ICC	0.220						
Conditional R^2	0.497						
Marginal R^2	0.277						

A generalized linear mixed model was estimated. β = Standardized regression coefficient. CI = Confidence Intervals. OR = Odds ratios based on standardized scores. P_{Holm} = One-tailed P-test, Holm-corrected for multiple comparisons. [1]Reference group = men. [2]Higher values mean a more right-leaning political orientation. [3]Reference group = Democrats. ICC = Intraclass correlation coefficients. R^2 = explained variance.

Table S4 | Conspiracy Theory 4: Standardized multi-level regression analysis predicting the likelihood that Twitter (currently X) engagements supported the conspiracy belief that governments and politicians are intentionally spreading false information, calculated on Twitter (currently X) engagements with sim > 0.25 ($N_{Participants}$ = 2,119, $N_{Engagements}$ = 565,274).

		95% CI			95% CI		
Variable	β	lower	upper	*OR*	lower	upper	P_{Holm}
(Intercept)	-4.11	-4.26	-3.95	0.02	0.01	0.02	<0.001
Followers on Twitter (currently X)	-0.08	-0.35	0.20	0.93	0.70	1.22	1.000
Following on Twitter (currently X)	0.09	-0.21	0.38	1.09	0.81	1.47	1.000
Age	0.38	0.30	0.45	1.46	1.35	1.57	<0.000
Education	-0.04	-0.11	0.02	0.96	0.90	1.02	1.000
Gender[1]: Woman	0.03	-0.10	0.17	1.03	0.91	1.18	1.000
Gender[1]: Other	0.24	-0.16	0.64	1.27	0.85	1.90	1.000
Political Orientation (linear)	-0.01	-0.11	0.09	0.99	0.89	1.09	1.000
Political Orientation (quadratic)	0.04	-0.02	0.11	1.04	0.98	1.12	1.000
Political Party[3]: Republican	-0.37	-0.62	-0.12	0.69	0.54	0.88	0.034
Political Party[3]: Independent	-0.19	-0.36	-0.02	0.83	0.70	0.98	0.225
Political Party[3]: Other	-0.40	-0.69	-0.11	0.67	0.50	0.90	0.063
Belief in False Information	0.09	0.01	0.17	1.10	1.01	1.19	0.221
Disbelief in True Information	0.03	-0.04	0.10	1.03	0.96	1.11	1.000
Conspiracy Mentality	0.07	-0.01	0.16	1.08	0.99	1.17	0.581
Narcissism	-0.06	-0.13	0.01	0.94	0.88	1.01	0.629
Denialism	-0.03	-0.11	0.06	0.97	0.89	1.06	1.000
Need for Chaos	0.02	-0.05	0.10	1.02	0.95	1.10	1.000
Belief in Information Reliability	-0.03	-0.10	0.04	0.97	0.90	1.04	1.000
Importance of Verifying Information	0.04	-0.03	0.11	1.04	0.97	1.12	1.000
Perceived Ability to Recognize Misinformation	0.06	-0.01	0.12	1.06	0.99	1.13	0.629
Adjusted ICC	0.17						
Unadjusted ICC	0.16						
Conditional R^2	0.21						
Marginal R^2	0.05						

A generalized linear mixed model was estimated. β = Standardized regression coefficient. CI = Confidence Intervals. OR = Odds ratios based on standardized scores. P_{Holm} = One-tailed P-test, Holm-corrected for multiple comparisons. [1]Reference group = men. [2]Higher values mean a more right-leaning political orientation. [3]Reference group = Democrats. ICC = Intraclass correlation coefficients. R^2 = explained variance.

Table S5 | Conspiracy Theory 5: Standardized multi-level regression analysis predicting the likelihood that Twitter (currently X) engagements supported the conspiracy belief that China intentionally spread the virus to hurt other countries, calculated on Twitter (currently X) engagements with sim > 0.25 ($N_{Participants}$ = 2,015, $N_{Engagements}$ = 291,514).

		95% CI			95% CI		
Variable	β	lower	upper	*OR*	lower	upper	P_{Holm}
(Intercept)	-8.61	-9.48	-7.74	0.00	0.00	0.00	0.000
Followers on Twitter (currently X)	-0.80	-2.09	0.50	0.45	0.12	1.65	1.000
Following on Twitter (currently X)	0.71	-0.46	1.88	2.03	0.63	6.54	1.000
Age	0.10	-0.22	0.42	1.10	0.80	1.51	1.000
Education	0.05	-0.24	0.34	1.05	0.78	1.40	1.000
Gender[1]: Woman	-0.21	-0.78	0.37	0.81	0.46	1.44	1.000
Gender[1]: Other	-1.25	-3.67	1.17	0.29	0.03	3.24	1.000
Political Orientation (linear)	0.33	-0.10	0.77	1.40	0.91	2.15	1.000
Political Orientation (quadratic)	-0.07	-0.35	0.21	0.93	0.71	1.23	1.000
Political Party[3]: Republican	0.07	-0.99	1.12	1.07	0.37	3.07	1.000
Political Party[3]: Independent	0.39	-0.44	1.23	1.48	0.64	3.42	1.000
Political Party[3]: Other	-0.16	-1.46	1.13	0.85	0.23	3.10	1.000
Belief in False Information	0.39	0.07	0.70	1.47	1.08	2.02	0.157
Disbelief in True Information	0.21	-0.08	0.51	1.24	0.92	1.67	1.000
Conspiracy Mentality	0.16	-0.22	0.53	1.17	0.80	1.70	1.000
Narcissism	-0.24	-0.54	0.05	0.78	0.58	1.06	1.000
Denialism	0.09	-0.30	0.48	1.09	0.74	1.61	1.000
Need for Chaos	-0.05	-0.39	0.29	0.95	0.68	1.34	1.000
Belief in Information Reliability	0.16	-0.12	0.45	1.18	0.89	1.56	1.000
Importance of Verifying Information	0.01	-0.27	0.30	1.01	0.76	1.35	1.000
Perceived Ability to Recognize Misinformation	-0.05	-0.33	0.23	0.95	0.72	1.26	1.000
Adjusted ICC	0.30						
Unadjusted ICC	0.24						
Conditional R^2	0.43						
Marginal R^2	0.19						

A generalized linear mixed model was estimated. β = Standardized regression coefficient. CI = Confidence Intervals. OR = Odds ratios based on standardized scores. P_{Holm} = One-tailed P-test, Holm-corrected for multiple comparisons. [1]Reference group = men. [2]Higher values mean a more right-leaning political orientation. [3]Reference group = Democrats. ICC = Intraclass correlation coefficients. R^2 = explained variance.

Table S6 | Conspiracy Theory 6: Standardized multi-level regression analysis predicting the likelihood that Twitter (currently X) engagements supported the conspiracy belief that the vaccines are unsafe or a means of population control, calculated on Twitter (currently X) engagements with sim > 0.25 ($N_{Participants}$ = 2,088 , $N_{Engagements}$ = 381,835).

		95% CI			95% CI		
Variable	β	lower	upper	*OR*	lower	upper	P_{Holm}
(Intercept)	-7.18	-7.63	-6.73	0.00	0.00	0.00	<0.001
Followers on Twitter (currently X)	0.02	-0.74	0.79	1.02	0.48	2.19	1.000
Following on Twitter (currently X)	-0.12	-0.85	0.60	0.88	0.43	1.83	1.000
Age	0.29	0.10	0.49	1.34	1.10	1.63	0.035
Education	-0.11	-0.28	0.07	0.90	0.76	1.07	1.000
Gender[1]: Woman	-0.43	-0.78	-0.09	0.65	0.46	0.92	0.131
Gender[1]: Other	-0.84	-2.01	0.32	0.43	0.13	1.38	0.938
Political Orientation (linear)	0.20	-0.05	0.45	1.22	0.96	1.56	0.776
Political Orientation (quadratic)	0.05	-0.11	0.22	1.06	0.89	1.25	1.000
Political Party[3]: Republican	-0.03	-0.66	0.59	0.97	0.52	1.81	1.000
Political Party[3]: Independent	0.34	-0.12	0.80	1.40	0.89	2.21	0.934
Political Party[3]: Other	0.26	-0.45	0.97	1.30	0.64	2.64	1.000
Belief in False Information	0.30	0.11	0.50	1.35	1.11	1.65	0.026
Disbelief in True Information	0.17	-0.01	0.35	1.18	0.99	1.42	0.534
Conspiracy Mentality	0.19	-0.04	0.41	1.21	0.96	1.51	0.754
Narcissism	-0.02	-0.20	0.16	0.98	0.82	1.18	1.000
Denialism	0.24	0.01	0.47	1.27	1.01	1.60	0.329
Need for Chaos	0.03	-0.16	0.22	1.03	0.85	1.24	1.000
Belief in Information Reliability	0.12	-0.06	0.30	1.13	0.94	1.35	1.000
Importance of Verifying Information	0.12	-0.07	0.31	1.12	0.93	1.36	1.000
Perceived Ability to Recognize Misinformation	0.06	-0.12	0.24	1.06	0.89	1.27	1.000
Adjusted ICC	0.38						
Unadjusted ICC	0.31						
Conditional R^2	0.48						
Marginal R^2	0.17						

A generalized linear mixed model was estimated. β = Standardized regression coefficient. CI = Confidence Intervals. OR = Odds ratios based on standardized scores. P_{Holm} = One-tailed P-test, Holm-corrected for multiple comparisons. [1]Reference group = men. [2]Higher values mean a more right-leaning political orientation. [3]Reference group = Democrats. ICC = Intraclass correlation coefficients. R^2 = explained variance.

Table S7 | Conspiracy Theory 1: Association between predictors and likelihood that Twitter (currently X) engagement supported conspiracy theory by engagement type (Separate models were estimated for each type).

Predictor / Engagement Type	Likes (*N* = 3,607,354)			Posts (*N* = 1,012,565)			Replies (*N* = 1,084,863)			Reposts (*N* = 2,008,724)		
	β	95% CI	P_{Holm}	β	95% CI	P_{Holm}	β	95% CI	P_{Holm}	β	95% CI	P_{Holm}
(Intercept)	-11.07	[-11.72, -10.43]	0.000	-15.04	[-17.63, -12.45]	0.000	-13.71	[-16.22, -11.2]	0.000	-9.25	[-9.82, -8.68]	0.000
Followers on Twitter (currently X)	0.01	[-0.77, 0.8]	1.000	-0.01	[-2.02, 2]	1.000	0.29	[-1.24, 1.82]	1.000	0.09	[-0.71, 0.88]	1.000
Following on Twitter (currently X)	0.00	[-0.86, 0.85]	1.000	0.08	[-2.2, 2.36]	1.000	-0.24	[-1.97, 1.49]	1.000	-0.19	[-0.97, 0.59]	1.000
Age	0.59	[0.34, 0.83]	0.000	1.05	[0.12, 1.99]	0.271	1.21	[0.42, 2]	0.026	0.67	[0.43, 0.91]	0.000
Education	0.27	[0.06, 0.48]	0.111	0.38	[-0.43, 1.18]	1.000	-0.04	[-0.67, 0.58]	1.000	0.24	[0.03, 0.46]	0.215
Gender[1]: Woman	-0.06	[-0.5, 0.37]	1.000	0.13	[-1.59, 1.85]	1.000	0.55	[-0.89, 1.98]	1.000	-0.02	[-0.46, 0.43]	1.000
Gender[1]: Other	-0.30	[-1.71, 1.12]	1.000	0.67	[-3.37, 4.71]	1.000	0.64	[-2.67, 3.95]	1.000	-0.02	[-1.12, 1.07]	1.000
Political Orientation (linear)	-0.18	[-0.5, 0.13]	1.000	-0.06	[-1.13, 1]	1.000	-0.01	[-0.98, 0.96]	1.000	-0.52	[-0.86, -0.18]	0.026
Political Orientation (quadratic)	0.26	[0.04, 0.47]	0.167	0.16	[-0.58, 0.9]	1.000	0.24	[-0.46, 0.95]	1.000	0.16	[-0.08, 0.4]	1.000
Political Party[3]: Republican	-0.23	[-1.04, 0.58]	1.000	-0.11	[-2.92, 2.7]	1.000	0.03	[-2.51, 2.58]	1.000	0.06	[-0.85, 0.96]	1.000
Political Party[3]: Independent	0.36	[-0.2, 0.92]	1.000	0.41	[-1.81, 2.64]	1.000	-0.29	[-2.2, 1.63]	1.000	0.22	[-0.32, 0.75]	1.000
Political Party[3]: Other	0.23	[-0.68, 1.14]	1.000	1.38	[-1.12, 3.89]	1.000	0.85	[-1.48, 3.18]	1.000	0.40	[-0.41, 1.2]	1.000
Belief in False Information	0.33	[0.07, 0.59]	0.111	-0.23	[-1.17, 0.71]	1.000	0.55	[-0.31, 1.4]	1.000	0.04	[-0.24, 0.31]	1.000
Disbelief in True Information	-0.05	[-0.29, 0.18]	1.000	0.39	[-0.43, 1.22]	1.000	-0.40	[-1.21, 0.4]	1.000	-0.21	[-0.46, 0.03]	0.736
Conspiracy Mentality	0.19	[-0.09, 0.47]	1.000	0.28	[-0.72, 1.29]	1.000	-0.10	[-0.91, 0.71]	1.000	0.05	[-0.22, 0.32]	1.000
Narcissism	-0.20	[-0.43, 0.03]	0.684	-0.38	[-1.27, 0.5]	1.000	0.13	[-0.6, 0.86]	1.000	-0.04	[-0.26, 0.19]	1.000
Denialism	0.16	[-0.13, 0.45]	1.000	-0.03	[-1.06, 1]	1.000	0.49	[-0.39, 1.37]	1.000	-0.04	[-0.32, 0.24]	1.000
Need for Chaos	-0.07	[-0.31, 0.17]	1.000	0.26	[-0.62, 1.14]	1.000	-0.38	[-1.26, 0.51]	1.000	-0.10	[-0.36, 0.17]	1.000
Belief in Information Reliability	0.05	[-0.18, 0.27]	1.000	0.65	[-0.22, 1.52]	1.000	0.16	[-0.53, 0.86]	1.000	-0.05	[-0.28, 0.18]	1.000
Importance of Verifying Information	0.04	[-0.2, 0.27]	1.000	0.60	[-0.41, 1.61]	1.000	-0.55	[-1.21, 0.11]	1.000	0.01	[-0.24, 0.25]	1.000
Perceived Ability to Recognize Misinformation	0.05	[-0.17, 0.27]	1.000	-0.18	[-0.94, 0.58]	1.000	0.23	[-0.51, 0.96]	1.000	0.17	[-0.06, 0.39]	1.000

A generalized linear mixed model was estimated. B = Unstandardized effect. CI = Confidence Intervals. OR = Odds ratios based on standardized scores. P_{Holm} = One-tailed P-test, Holm-corrected for multiple comparisons. [1]Reference group = men. [2]Higher values mean a more right-leaning political orientation. [3]Reference group = Democrats.

Table S8 | Conspiracy Theory 2: Association between predictors and likelihood that Twitter (currently X) engagement supported conspiracy theory by engagement type (Separate models were estimated for each type).

Predictor / Engagement Type	Likes (*N* = 3,607,354)			Posts (*N* = 1,012,565)			Replies (*N* = 1,084,863)			Reposts (*N* = 2,008,724)		
	β	95% CI	P_{Holm}	β	95% CI	P_{Holm}	β	95% CI	P_{Holm}	β	95% CI	P_{Holm}
(Intercept)	-8.92	[-9.26, -8.59]	0.000	-10.50	[-11.58, -9.42]	0.000	-9.74	[-10.5, -8.99]	0.000	-7.99	[-8.42, -7.55]	0.000
Followers on Twitter (currently X)	-0.07	[-0.66, 0.51]	1.000	-0.17	[-1.08, 0.74]	1.000	-0.30	[-1.13, 0.53]	1.000	0.34	[-0.26, 0.94]	1.000
Following on Twitter (currently X)	-0.09	[-0.64, 0.47]	1.000	0.14	[-0.87, 1.15]	1.000	0.37	[-0.55, 1.29]	1.000	-0.54	[-1.13, 0.06]	0.659
Age	0.69	[0.54, 0.83]	0.000	1.03	[0.63, 1.43]	0.000	0.90	[0.59, 1.21]	0.000	0.85	[0.65, 1.05]	0.000
Education	0.10	[-0.03, 0.22]	0.919	0.06	[-0.29, 0.42]	1.000	0.07	[-0.2, 0.33]	1.000	0.12	[-0.06, 0.29]	1.000
Gender[1]: Woman	0.00	[-0.26, 0.26]	1.000	-0.78	[-1.49, -0.07]	0.298	-0.21	[-0.75, 0.33]	1.000	0.08	[-0.27, 0.43]	1.000
Gender[1]: Other	-0.37	[-1.27, 0.53]	1.000	-0.16	[-2.39, 2.06]	1.000	-0.55	[-2.37, 1.27]	1.000	-0.07	[-1.09, 0.94]	1.000
Political Orientation (linear)	0.10	[-0.09, 0.29]	1.000	0.13	[-0.34, 0.61]	1.000	0.05	[-0.34, 0.44]	1.000	-0.06	[-0.31, 0.2]	1.000
Political Orientation (quadratic)	0.19	[0.06, 0.32]	0.028	0.23	[-0.11, 0.56]	1.000	0.21	[-0.05, 0.48]	0.986	0.18	[0, 0.35]	0.426
Political Party[3]: Republican	0.28	[-0.16, 0.72]	1.000	-0.53	[-1.72, 0.66]	1.000	0.05	[-0.9, 1]	1.000	-0.14	[-0.78, 0.51]	1.000
Political Party[3]: Independent	0.50	[0.16, 0.83]	0.031	-0.08	[-1.01, 0.84]	1.000	0.22	[-0.49, 0.93]	1.000	0.06	[-0.38, 0.5]	1.000
Political Party[3]: Other	0.24	[-0.33, 0.82]	1.000	0.25	[-1.09, 1.59]	1.000	0.12	[-1, 1.23]	1.000	-0.59	[-1.35, 0.18]	0.984
Belief in False Information	0.34	[0.19, 0.49]	0.000	0.17	[-0.24, 0.58]	1.000	0.37	[0.06, 0.67]	0.177	0.23	[0.03, 0.44]	0.262
Disbelief in True Information	-0.04	[-0.17, 0.1]	1.000	0.01	[-0.36, 0.38]	1.000	-0.01	[-0.3, 0.27]	1.000	-0.03	[-0.21, 0.16]	1.000
Conspiracy Mentality	-0.02	[-0.18, 0.15]	1.000	0.11	[-0.32, 0.54]	1.000	0.12	[-0.21, 0.44]	1.000	0.04	[-0.18, 0.25]	1.000
Narcissism	-0.08	[-0.21, 0.05]	1.000	-0.24	[-0.6, 0.13]	1.000	-0.19	[-0.47, 0.09]	1.000	-0.11	[-0.3, 0.07]	1.000
Denialism	0.27	[0.09, 0.44]	0.025	0.32	[-0.15, 0.79]	1.000	0.21	[-0.15, 0.57]	1.000	0.15	[-0.07, 0.38]	1.000
Need for Chaos	-0.22	[-0.36, -0.07]	0.026	-0.04	[-0.43, 0.35]	1.000	-0.17	[-0.47, 0.13]	1.000	-0.15	[-0.35, 0.04]	0.967
Belief in Information Reliability	-0.05	[-0.18, 0.08]	1.000	0.22	[-0.16, 0.59]	1.000	0.30	[0.01, 0.58]	0.381	-0.02	[-0.2, 0.17]	1.000
Importance of Verifying Information	0.01	[-0.13, 0.16]	1.000	0.14	[-0.25, 0.53]	1.000	-0.22	[-0.5, 0.07]	1.000	0.14	[-0.05, 0.33]	1.000
Perceived Ability to Recognize Misinformation	0.18	[0.05, 0.31]	0.053	0.08	[-0.27, 0.42]	1.000	0.04	[-0.24, 0.32]	1.000	0.12	[-0.06, 0.3]	1.000

A generalized linear mixed model was estimated. B = Unstandardized effect. CI = Confidence Intervals. OR = Odds ratios based on standardized scores. P_{Holm} = One-tailed P-test, Holm-corrected for multiple comparisons. [1]Reference group = men. [2]Higher values mean a more right-leaning political orientation. [3]Reference group = Democrats.

Table S9 | Conspiracy Theory 3: Association between predictors and likelihood that Twitter (currently X) engagement supported conspiracy theory by engagement type (Separate models were estimated for each type).

Predictor / Engagement Type	Likes (*N* = 3,607,354)			Posts (*N* = 1,012,565)			Replies (*N* = 1,084,863)			Reposts (*N* = 2,008,724)		
	β	95% CI	P_{Holm}	β	95% CI	P_{Holm}	β	95% CI	P_{Holm}	β	95% CI	P_{Holm}
(Intercept)	-11.74	[-12.55, -10.92]	0.000	-14.81	[-17.44, -12.18]	0.000	-13.36	[-15.91, -10.81]	0.000	-11.44	[-12.75, -10.14]	0.000
Followers on Twitter (currently X)	-0.66	[-1.95, 0.62]	1.000	-0.10	[-2, 1.81]	1.000	-0.51	[-2.69, 1.68]	1.000	0.37	[-0.88, 1.63]	1.000
Following on Twitter (currently X)	0.59	[-0.53, 1.71]	1.000	-0.34	[-2.44, 1.75]	1.000	0.61	[-1.75, 2.98]	1.000	-0.62	[-1.84, 0.6]	1.000
Age	0.67	[0.39, 0.96]	0.000	1.14	[0.18, 2.11]	0.206	0.60	[-0.22, 1.42]	1.000	0.77	[0.32, 1.23]	0.008
Education	0.06	[-0.2, 0.31]	1.000	0.22	[-0.58, 1.03]	1.000	0.17	[-0.51, 0.85]	1.000	0.07	[-0.32, 0.47]	1.000
Gender[1]: Woman	-0.36	[-0.86, 0.15]	1.000	-0.81	[-2.45, 0.82]	1.000	-0.48	[-1.86, 0.9]	1.000	-0.67	[-1.5, 0.17]	0.993
Gender[1]: Other	-0.21	[-2.28, 1.86]	1.000	-0.48	[-5.61, 4.64]	1.000	-15.65	[-8497.13, 8465.83]	1.000	0.42	[-1.9, 2.74]	1.000
Political Orientation (linear)	0.52	[0.12, 0.93]	0.108	-0.07	[-1.12, 0.98]	1.000	0.34	[-0.71, 1.39]	1.000	0.88	[0.23, 1.52]	0.074
Political Orientation (quadratic)	0.00	[-0.26, 0.26]	1.000	0.39	[-0.32, 1.11]	1.000	-0.30	[-1.01, 0.42]	1.000	-0.27	[-0.69, 0.16]	1.000
Political Party[3]: Republican	0.41	[-0.48, 1.3]	1.000	0.13	[-2.65, 2.91]	1.000	0.59	[-1.83, 3.02]	1.000	0.36	[-1.06, 1.78]	1.000
Political Party[3]: Independent	0.83	[0.1, 1.55]	0.211	0.86	[-1.46, 3.18]	1.000	0.52	[-1.41, 2.45]	1.000	0.61	[-0.56, 1.78]	1.000
Political Party[3]: Other	0.25	[-1, 1.5]	1.000	0.91	[-2.07, 3.9]	1.000	-0.44	[-3.65, 2.76]	1.000	0.35	[-1.4, 2.09]	1.000
Belief in False Information	0.35	[0.07, 0.63]	0.136	0.48	[-0.45, 1.4]	1.000	0.66	[-0.08, 1.4]	0.804	0.28	[-0.15, 0.72]	1.000
Disbelief in True Information	0.14	[-0.12, 0.4]	1.000	0.12	[-0.78, 1.01]	1.000	0.42	[-0.27, 1.11]	1.000	-0.02	[-0.45, 0.41]	1.000
Conspiracy Mentality	0.12	[-0.22, 0.45]	1.000	0.11	[-0.89, 1.11]	1.000	0.00	[-0.8, 0.81]	1.000	0.15	[-0.35, 0.65]	1.000
Narcissism	-0.24	[-0.5, 0.02]	0.550	-0.22	[-1.09, 0.65]	1.000	-0.47	[-1.15, 0.21]	1.000	-0.41	[-0.86, 0.04]	0.660
Denialism	0.31	[-0.03, 0.65]	0.550	0.19	[-0.83, 1.2]	1.000	0.70	[-0.28, 1.67]	1.000	0.41	[-0.13, 0.96]	1.000
Need for Chaos	-0.32	[-0.62, -0.01]	0.339	-0.18	[-1.1, 0.74]	1.000	-0.18	[-0.98, 0.61]	1.000	-0.07	[-0.52, 0.38]	1.000
Belief in Information Reliability	-0.09	[-0.35, 0.17]	1.000	0.28	[-0.58, 1.13]	1.000	-0.12	[-0.79, 0.55]	1.000	0.08	[-0.35, 0.5]	1.000
Importance of Verifying Information	-0.04	[-0.3, 0.23]	1.000	-0.16	[-1.07, 0.74]	1.000	0.25	[-0.51, 1.01]	1.000	0.00	[-0.44, 0.44]	1.000
Perceived Ability to Recognize Misinformation	0.16	[-0.09, 0.41]	1.000	0.16	[-0.62, 0.94]	1.000	0.23	[-0.48, 0.94]	1.000	0.02	[-0.4, 0.44]	1.000

A generalized linear mixed model was estimated. B = Unstandardized effect. CI = Confidence Intervals. OR = Odds ratios based on standardized scores. P_{Holm} = One-tailed P-test, Holm-corrected for multiple comparisons. [1]Reference group = men. [2]Higher values mean a more right-leaning political orientation. [3]Reference group = Democrats.

Table S10 | Conspiracy Theory 4: Association between predictors and likelihood that Twitter (currently X) engagement supported conspiracy theory by engagement type (Separate models were estimated for each type).

Predictor / Engagement Type	Likes (*N* = 3,607,354)			Posts (*N* = 1,012,565)			Replies (*N* = 1,084,863)			Reposts (*N* = 2,008,724)		
	β	95% CI	P_{Holm}	β	95% CI	P_{Holm}	β	95% CI	P_{Holm}	β	95% CI	P_{Holm}
(Intercept)	-8.57	[-8.89, -8.25]	0.000	-10.21	[-11.22, -9.21]	0.000	-9.09	[-9.71, -8.48]	0.000	-7.27	[-7.65, -6.89]	0.000
Followers on Twitter (currently X)	-0.06	[-0.62, 0.49]	1.000	-0.16	[-1.05, 0.72]	1.000	-0.38	[-1.16, 0.41]	1.000	0.19	[-0.37, 0.75]	1.000
Following on Twitter (currently X)	-0.06	[-0.6, 0.48]	1.000	0.13	[-0.86, 1.13]	1.000	0.40	[-0.46, 1.26]	1.000	-0.34	[-0.9, 0.22]	1.000
Age	0.82	[0.68, 0.97]	0.000	1.22	[0.84, 1.61]	0.000	1.24	[0.97, 1.52]	0.000	1.01	[0.84, 1.19]	0.000
Education	0.09	[-0.03, 0.21]	0.995	0.18	[-0.14, 0.51]	1.000	-0.01	[-0.24, 0.23]	1.000	0.08	[-0.08, 0.23]	1.000
Gender[1]: Woman	0.08	[-0.17, 0.33]	1.000	-0.56	[-1.23, 0.11]	0.988	0.06	[-0.43, 0.54]	1.000	0.20	[-0.11, 0.52]	1.000
Gender[1]: Other	-0.19	[-1.03, 0.65]	1.000	0.13	[-1.86, 2.11]	1.000	0.17	[-1.29, 1.62]	1.000	0.45	[-0.4, 1.3]	1.000
Political Orientation (linear)	-0.01	[-0.19, 0.18]	1.000	-0.21	[-0.68, 0.26]	1.000	-0.01	[-0.36, 0.35]	1.000	-0.25	[-0.48, -0.02]	0.289
Political Orientation (quadratic)	0.22	[0.09, 0.34]	0.008	0.19	[-0.14, 0.52]	1.000	0.22	[-0.03, 0.46]	0.823	0.23	[0.06, 0.39]	0.068
Political Party[3]: Republican	-0.18	[-0.62, 0.26]	1.000	-0.87	[-2.06, 0.33]	1.000	-0.65	[-1.55, 0.24]	1.000	-0.81	[-1.42, -0.2]	0.087
Political Party[3]: Independent	0.21	[-0.11, 0.53]	1.000	-0.37	[-1.24, 0.49]	1.000	-0.51	[-1.14, 0.13]	1.000	-0.27	[-0.66, 0.12]	1.000
Political Party[3]: Other	-0.17	[-0.74, 0.4]	1.000	0.33	[-0.89, 1.56]	1.000	-0.15	[-1.12, 0.83]	1.000	-0.56	[-1.2, 0.09]	0.690
Belief in False Information	0.28	[0.13, 0.43]	0.002	0.12	[-0.29, 0.53]	1.000	0.14	[-0.16, 0.43]	1.000	0.09	[-0.1, 0.28]	1.000
Disbelief in True Information	-0.11	[-0.25, 0.02]	0.799	0.00	[-0.35, 0.35]	1.000	-0.05	[-0.31, 0.22]	1.000	-0.01	[-0.17, 0.16]	1.000
Conspiracy Mentality	0.08	[-0.08, 0.24]	1.000	0.12	[-0.29, 0.52]	1.000	0.10	[-0.19, 0.39]	1.000	0.02	[-0.18, 0.21]	1.000
Narcissism	-0.10	[-0.23, 0.03]	0.878	-0.12	[-0.46, 0.23]	1.000	-0.20	[-0.45, 0.06]	1.000	-0.10	[-0.26, 0.06]	1.000
Denialism	0.07	[-0.1, 0.23]	1.000	-0.08	[-0.51, 0.35]	1.000	-0.03	[-0.34, 0.28]	1.000	-0.05	[-0.26, 0.15]	1.000
Need for Chaos	-0.14	[-0.29, 0]	0.356	0.02	[-0.36, 0.41]	1.000	0.03	[-0.25, 0.31]	1.000	0.01	[-0.16, 0.18]	1.000
Belief in Information Reliability	-0.04	[-0.17, 0.09]	1.000	0.08	[-0.27, 0.43]	1.000	0.06	[-0.2, 0.31]	1.000	-0.10	[-0.26, 0.07]	1.000
Importance of Verifying Information	0.04	[-0.1, 0.18]	1.000	0.12	[-0.26, 0.49]	1.000	0.00	[-0.27, 0.27]	1.000	0.13	[-0.05, 0.3]	1.000
Perceived Ability to Recognize Misinformation	0.16	[0.03, 0.29]	0.105	0.18	[-0.16, 0.52]	1.000	-0.02	[-0.27, 0.22]	1.000	0.18	[0.02, 0.34]	0.218

A generalized linear mixed model was estimated. B = Unstandardized effect. CI = Confidence Intervals. OR = Odds ratios based on standardized scores. P_{Holm} = One-tailed P-test, Holm-corrected for multiple comparisons. [1]Reference group = men. [2]Higher values mean a more right-leaning political orientation. [3]Reference group = Democrats.

Table S11 | Conspiracy Theory 5: Association between predictors and likelihood that Twitter (currently X) engagement supported conspiracy theory by engagement type (Separate models were estimated for each type).

Predictor / Engagement Type	Likes (*N* = 3,607,354)			Posts (*N* = 1,012,565)			Replies (*N* = 1,084,863)			Reposts (*N* = 2,008,724)		
	β	95% CI	P_{Holm}	β	95% CI	P_{Holm}	β	95% CI	P_{Holm}	β	95% CI	P_{Holm}
(Intercept)	-16.45	[-19.16, -13.74]	0.000	-16.46	[-21.09, -11.82]	0.000	-16.58	[-21.44, -11.71]	0.000	-13.54	[-15.97, -11.11]	0.000
Followers on Twitter (currently X)	-0.55	[-4.34, 3.23]	1.000	-0.31	[-5.22, 4.61]	1.000	-1.52	[-5.12, 2.09]	1.000	-0.15	[-2.65, 2.35]	1.000
Following on Twitter (currently X)	0.25	[-2.94, 3.44]	1.000	-0.51	[-6.13, 5.11]	1.000	1.91	[-2.18, 5.99]	1.000	-0.27	[-2.61, 2.07]	1.000
Age	0.61	[-0.28, 1.51]	1.000	0.03	[-1.92, 1.99]	1.000	-0.06	[-1.56, 1.44]	1.000	1.02	[0.23, 1.81]	0.112
Education	0.12	[-0.65, 0.9]	1.000	-0.02	[-1.71, 1.66]	1.000	0.93	[-0.3, 2.17]	1.000	-0.07	[-0.75, 0.62]	1.000
Gender[1]: Woman	-0.26	[-1.8, 1.29]	1.000	-1.55	[-5, 1.89]	1.000	0.54	[-2.07, 3.15]	1.000	0.15	[-1.26, 1.56]	1.000
Gender[1]: Other	-14.03	[-5297.62, 5269.56]	1.000	-14.57	[-8597.7, 8568.57]	1.000	-13.62	[-7959.51, 7932.26]	1.000	-0.83	[-5.72, 4.06]	1.000
Political Orientation (linear)	0.05	[-1.11, 1.22]	1.000	0.56	[-1.76, 2.88]	1.000	-0.33	[-2.22, 1.55]	1.000	0.73	[-0.28, 1.73]	1.000
Political Orientation (quadratic)	0.15	[-0.6, 0.9]	1.000	0.28	[-1.23, 1.8]	1.000	-0.19	[-1.46, 1.08]	1.000	-0.09	[-0.76, 0.58]	1.000
Political Party[3]: Republican	0.74	[-2.06, 3.55]	1.000	-0.41	[-6.02, 5.19]	1.000	1.15	[-3.48, 5.77]	1.000	-0.64	[-2.97, 1.7]	1.000
Political Party[3]: Independent	0.76	[-1.66, 3.17]	1.000	0.80	[-3.85, 5.45]	1.000	0.79	[-2.8, 4.38]	1.000	0.07	[-1.75, 1.89]	1.000
Political Party[3]: Other	1.24	[-2.06, 4.53]	1.000	-0.29	[-7.51, 6.92]	1.000	0.10	[-5.44, 5.64]	1.000	0.19	[-2.49, 2.87]	1.000
Belief in False Information	0.33	[-0.5, 1.17]	1.000	0.24	[-1.56, 2.05]	1.000	0.66	[-0.7, 2.03]	1.000	0.78	[0.03, 1.54]	0.403
Disbelief in True Information	0.30	[-0.47, 1.06]	1.000	0.16	[-1.52, 1.84]	1.000	0.79	[-0.44, 2.02]	1.000	-0.07	[-0.79, 0.66]	1.000
Conspiracy Mentality	0.44	[-0.59, 1.46]	1.000	0.34	[-1.71, 2.39]	1.000	0.53	[-1.14, 2.2]	1.000	-0.05	[-0.86, 0.77]	1.000
Narcissism	0.00	[-0.81, 0.8]	1.000	0.08	[-1.56, 1.72]	1.000	-0.73	[-1.99, 0.53]	1.000	-0.48	[-1.23, 0.27]	1.000
Denialism	0.19	[-0.87, 1.26]	1.000	-0.08	[-2.11, 1.95]	1.000	0.44	[-1.38, 2.26]	1.000	0.09	[-0.78, 0.95]	1.000
Need for Chaos	-0.22	[-1.01, 0.57]	1.000	-0.44	[-2.5, 1.62]	1.000	-0.36	[-1.75, 1.04]	1.000	-0.15	[-0.96, 0.66]	1.000
Belief in Information Reliability	0.35	[-0.44, 1.13]	1.000	0.12	[-1.52, 1.77]	1.000	0.30	[-0.98, 1.58]	1.000	0.28	[-0.42, 0.97]	1.000
Importance of Verifying Information	-0.22	[-0.99, 0.54]	1.000	0.37	[-1.66, 2.4]	1.000	0.41	[-0.94, 1.76]	1.000	-0.14	[-0.87, 0.59]	1.000
Perceived Ability to Recognize Misinformation	0.13	[-0.65, 0.91]	1.000	-0.14	[-1.72, 1.44]	1.000	-0.51	[-1.76, 0.73]	1.000	0.28	[-0.42, 0.99]	1.000

A generalized linear mixed model was estimated. B = Unstandardized effect. CI = Confidence Intervals. OR = Odds ratios based on standardized scores. P_{Holm} = One-tailed P-test, Holm-corrected for multiple comparisons. [1]Reference group = men. [2]Higher values mean a more right-leaning political orientation. [3]Reference group = Democrats.

Table S12 | Conspiracy Theory 6: Association between predictors and likelihood that Twitter (currently X) engagement supported conspiracy theory by engagement type (Separate models were estimated for each type).

Predictor / Engagement Type	Likes (*N* = 3,607,354)			Posts (*N* = 1,012,565)			Replies (*N* = 1,084,863)			Reposts (*N* = 2,008,724)		
	β	95% CI	P_{Holm}	β	95% CI	P_{Holm}	β	95% CI	P_{Holm}	β	95% CI	P_{Holm}
(Intercept)	-12.12	[-13.03, -11.22]	0.000	-12.95	[-15.33, -10.57]	0.000	-13.82	[-16.09, -11.54]	0.000	-11.48	[-12.65, -10.31]	0.000
Followers on Twitter (currently X)	-0.54	[-1.84, 0.76]	1.000	-0.48	[-2.88, 1.93]	1.000	-0.23	[-2.28, 1.83]	1.000	0.64	[-0.35, 1.62]	1.000
Following on Twitter (currently X)	0.01	[-1.18, 1.2]	1.000	-1.24	[-3.58, 1.09]	1.000	-0.02	[-2.29, 2.25]	1.000	-0.63	[-1.72, 0.45]	1.000
Age	0.78	[0.48, 1.09]	0.000	0.79	[0.07, 1.51]	0.325	0.92	[0.07, 1.77]	0.320	0.77	[0.33, 1.22]	0.007
Education	0.11	[-0.15, 0.38]	1.000	-0.26	[-0.88, 0.37]	1.000	-0.03	[-0.73, 0.67]	1.000	0.08	[-0.32, 0.49]	1.000
Gender[1]: Woman	-0.19	[-0.72, 0.34]	1.000	-1.15	[-2.39, 0.09]	0.631	-0.56	[-2.11, 0.99]	1.000	-0.05	[-0.88, 0.78]	1.000
Gender[1]: Other	-1.95	[-4.94, 1.04]	1.000	-1.94	[-5.94, 2.07]	1.000	1.16	[-2.2, 4.52]	1.000	-0.86	[-3.36, 1.63]	1.000
Political Orientation (linear)	0.14	[-0.24, 0.51]	1.000	0.23	[-0.52, 0.99]	1.000	0.03	[-1.03, 1.09]	1.000	0.00	[-0.55, 0.55]	1.000
Political Orientation (quadratic)	0.20	[-0.06, 0.46]	1.000	0.47	[-0.06, 0.99]	0.694	0.17	[-0.53, 0.87]	1.000	0.01	[-0.37, 0.39]	1.000
Political Party[3]: Republican	0.03	[-0.89, 0.96]	1.000	-1.32	[-3.35, 0.71]	1.000	-0.05	[-2.56, 2.46]	1.000	-0.25	[-1.69, 1.18]	1.000
Political Party[3]: Independent	0.77	[0.06, 1.48]	0.298	-0.44	[-2.21, 1.32]	1.000	-0.38	[-2.46, 1.7]	1.000	0.23	[-0.77, 1.23]	1.000
Political Party[3]: Other	0.83	[-0.29, 1.94]	1.000	1.01	[-0.82, 2.83]	1.000	-0.16	[-2.89, 2.57]	1.000	0.37	[-1.16, 1.91]	1.000
Belief in False Information	0.42	[0.12, 0.72]	0.057	0.68	[0.01, 1.34]	0.442	0.93	[0.13, 1.73]	0.231	0.34	[-0.11, 0.78]	1.000
Disbelief in True Information	0.10	[-0.18, 0.38]	1.000	0.23	[-0.41, 0.88]	1.000	0.35	[-0.39, 1.09]	1.000	0.38	[-0.03, 0.78]	0.598
Conspiracy Mentality	0.22	[-0.13, 0.57]	1.000	0.17	[-0.55, 0.9]	1.000	0.03	[-0.81, 0.87]	1.000	0.23	[-0.27, 0.73]	1.000
Narcissism	-0.06	[-0.34, 0.21]	1.000	-0.17	[-0.78, 0.44]	1.000	-0.22	[-0.98, 0.53]	1.000	-0.23	[-0.67, 0.2]	1.000
Denialism	0.31	[-0.05, 0.67]	0.794	0.30	[-0.47, 1.07]	1.000	0.19	[-0.74, 1.12]	1.000	0.57	[0.05, 1.1]	0.317
Need for Chaos	-0.12	[-0.4, 0.16]	1.000	0.11	[-0.5, 0.71]	1.000	-0.04	[-0.82, 0.73]	1.000	-0.20	[-0.62, 0.22]	1.000
Belief in Information Reliability	0.12	[-0.15, 0.39]	1.000	0.16	[-0.47, 0.79]	1.000	0.18	[-0.58, 0.93]	1.000	0.40	[-0.02, 0.81]	0.577
Importance of Verifying Information	0.10	[-0.2, 0.4]	1.000	0.54	[-0.21, 1.29]	1.000	-0.16	[-0.91, 0.59]	1.000	0.09	[-0.36, 0.53]	1.000
Perceived Ability to Recognize Misinformation	0.21	[-0.07, 0.48]	1.000	-0.02	[-0.6, 0.56]	1.000	-0.09	[-0.83, 0.65]	1.000	0.05	[-0.36, 0.47]	1.000

A generalized linear mixed model was estimated. B = Unstandardized effect. CI = Confidence Intervals. OR = Odds ratios based on standardized scores. P_{Holm} = One-tailed P-test, Holm-corrected for multiple comparisons. [1]Reference group = men. [2]Higher values mean a more right-leaning political orientation. [3]Reference group = Democrats.

Figure S2 | Correlations Between Self-Report Measures.

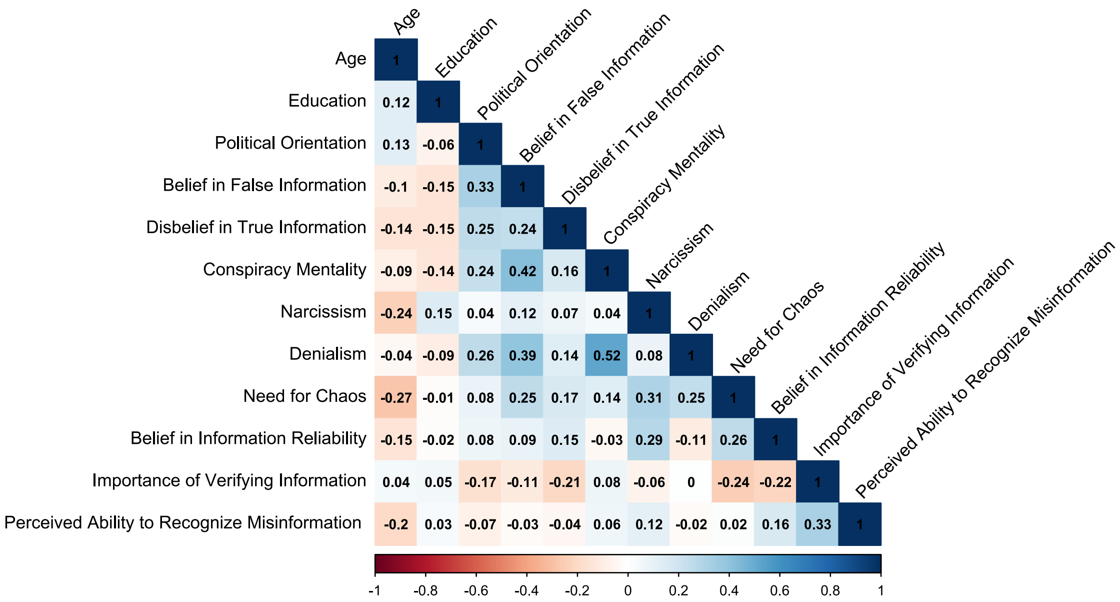

Pearson’s correlations are presented. *rs* ≥ 0.04 are significant at *P* < .05. All *rs* ≥ 0.05 are significant at *P* ≤ .001. *P*-values are two-tailed. For exact P-values, see Table S13. Ns = see Table S14.

Table S13 | Two-tailed P-values for correlations shown in Figure S2.

	1	2	3	4	5	6	7	8	9	10	11	12
**1 Age**		<0.001	<0.001	<0.001	<0.001	<0.001	<0.001	0.046	<0.001	<0.001	0.037	<0.001
**2 Education**			0.002	<0.001	<0.001	<0.001	<0.001	<0.001	0.494	0.411	0.012	0.085
**3 Political Orientation**				<0.001	<0.001	<0.001	0.073	<0.001	<0.001	<0.001	<0.001	<0.001
**4 Belief in False Information**					<0.001	<0.001	<0.001	<0.001	<0.001	<0.001	<0.001	0.141
**5 Disbelief in True Information**						<0.001	<0.001	<0.001	<0.001	<0.001	<0.001	0.031
**6 Conspiracy Mentality**							0.027	<0.001	<0.001	0.088	<0.001	0.002
**7 Narcissism**								<0.001	<0.001	<0.001	0.001	<0.001
**8 Denialism**									<0.001	<0.001	0.828	0.269
**9 Need for Chaos**										<0.001	<0.001	0.428
**10 Belief in Information Reliability**											<0.001	<0.001
**11 Importance of Verifying Information**												<0.001
**12 Perceived Ability to Recognize Misinformation**												

Table S14 | N for correlation tests shown in Figure S2.

	1	2	3	4	5	6	7	8	9	10	11	12
**1 Age**		2497	2499	2466	2466	2499	2499	2499	2499	2498	2498	2499
**2 Education**			2504	2471	2471	2504	2504	2504	2504	2503	2503	2504
**3 Political Orientation**				2473	2473	2506	2506	2506	2506	2505	2505	2506
**4 Belief in False Information**					2471	2473	2473	2473	2473	2472	2472	2473
**5 Disbelief in True Information**						2473	2473	2473	2473	2472	2472	2473
**6 Conspiracy Mentality**							2506	2506	2506	2505	2505	2506
**7 Narcissism**								2506	2506	2505	2505	2506
**8 Denialism**									2506	2505	2505	2506
**9 Need for Chaos**										2505	2505	2506
**10 Belief in Information Reliability**											2504	2505
**11 Importance of Verifying Information**												2505
**12 Perceived Ability to Recognize Misinformation**												
