# Supplementary material for: Leveraging artificial intelligence to identify the psychological factors associated with conspiracy theory beliefs online
Source: Nat Commun. 2024 Aug 29;15:7497. doi: 10.1038/s41467-024-51740-9 (PMC11362279; doi:10.1038/s41467-024-51740-9)
Supplement: Supplementary file 3 — Reporting Summary [file 41467_2024_51740_MOESM3_ESM.pdf]

nature portfolio

Corresponding author(s): Jonas R. Kunst

Last updated by author(s): Jul 3, 2023

Reporting Summary

Nature Portfolio wishes to improve the reproducibility of the work that we publish. This form provides structure for consistency and transparency in reporting. For further information on Nature Portfolio policies, see our Editorial Policies and the Editorial Policy Checklist.

Statistics

For all statistical analyses, confirm that the following items are present in the figure legend, table legend, main text, or Methods section.

n/a	Confirmed	
☐	☒	The exact sample size (*n*) for each experimental group/condition, given as a discrete number and unit of measurement
☐	☒	A statement on whether measurements were taken from distinct samples or whether the same sample was measured repeatedly
☐	☒	The statistical test(s) used AND whether they are one- or two-sided<br>*Only common tests should be described solely by name; describe more complex techniques in the Methods section.*
☐	☒	A description of all covariates tested
☐	☒	A description of any assumptions or corrections, such as tests of normality and adjustment for multiple comparisons
☐	☒	A full description of the statistical parameters including central tendency (e.g. means) or other basic estimates (e.g. regression coefficient) AND variation (e.g. standard deviation) or associated estimates of uncertainty (e.g. confidence intervals)
☐	☒	For null hypothesis testing, the test statistic (e.g. *F*, *t*, *r*) with confidence intervals, effect sizes, degrees of freedom and *P* value noted<br>*Give P values as exact values whenever suitable.*
☒	☐	For Bayesian analysis, information on the choice of priors and Markov chain Monte Carlo settings
☐	☒	For hierarchical and complex designs, identification of the appropriate level for tests and full reporting of outcomes
☐	☒	Estimates of effect sizes (e.g. Cohen's *d*, Pearson's *r*), indicating how they were calculated

Our web collection on statistics for biologists contains articles on many of the points above.

Software and code

Policy information about availability of computer code

Data collection	Qualtrics, Twitter (currently X) Academic API
Data analysis	For similarity classification:<br>sentence transformer model (sentence-transformers/all-mpnet-base-v2)<br><br>For conspiracy theory support classification via GPT:<br>gpt-3.5-turbo-0125<br><br>For main analysis:<br>We used the glmmTMB package v. 1.1.9 to estimate binomial multi-level generalized linear models models in R 4.2.274 to test for the effects of the psychological variables (level 2) on the X engagement estimates (level 1). The performance package v. 0.10.1 was used to estimate intraclass correlation coefficients (ICC) and explained variance (R2). Importantly, to enable the comparison of effect sizes, all predictors except for categorical factors (e.g., gender), were centered and standardized. In addition to the standardized coefficients, we report odds ratios for interpretability. Ggplot v. 3.4.0 and Ggeffects 1.1.5 were used for graphs. Correlations did not suggest muti-collinearity (see Figure 2). All reported P-values in the main models are one-tailed as predictions were unidirectional. They were Holm-corrected to adjust for multiple tests. The assumptions underlying the models were evaluated using version 0.4.6 of the DHARMA package.

For manuscripts utilizing custom algorithms or software that are central to the research but not yet described in published literature, software must be made available to editors and reviewers. We strongly encourage code deposition in a community repository (e.g. GitHub). See the Nature Portfolio guidelines for submitting code & software for further information.

Data

Policy information about availability of data

All manuscripts must include a data availability statement. This statement should provide the following information, where applicable:

- Accession codes, unique identifiers, or web links for publicly available datasets
- A description of any restrictions on data availability
- For clinical datasets or third party data, please ensure that the statement adheres to our policy

Data Availability
The data utilized in this study, required for replicating our statistical analyses, have been deposited in anonymized form in the Open Science Foundation database at https://doi.org/10.17605/OSF.IO/XPVFZ. The raw Twitter (currently X) data are available under restricted access in adherence to GDPR and the stipulations of the ethics approval and the data management board (JRK, JP, RR, MM). To maintain transparency and reproducibility, we offer remote access to the data (safe haven data sharing model). Access can be obtained by contacting the corresponding author. We aim to process and respond to requests within a month.

Code Availability
The code required for replicating our results can be accessed at https://doi.org/10.17605/OSF.IO/XPVFZ

Research involving human participants, their data, or biological material

Policy information about studies with human participants or human data. See also policy information about sex, gender (identity/presentation), and sexual orientation and race, ethnicity and racism.

Reporting on sex and gender	Gender was based on participants' self-reported responses and allowed participants to indicate a third option/non-binary/ "other", or to not respond. Gender was analyzed as predictor in the main analyses.<br><br>Gender in % of the sample:<br>Man 45.80<br>Woman 52.00<br>Third gender / nonbinary / other 2.10
Reporting on race, ethnicity, or other socially relevant groupings	Ethnicity was assessed via self-report to describe the sample, informing generalizations. Ethnicity is not used as predictor/ covariate in any models. It is also not used as a proxy for other variables.<br><br>Ethnicity in %<br>White 77.60<br>Black or African American 12.40<br>American Indian or Alaska Native 1.36<br>Asian 4.27<br>Native Hawaiian or Pacific Islander 0.32<br>Other 4.07
Population characteristics	The population were Twitter (currently X) users from the U.S. This population was chosen as our goal was to explain large scale behavior on one of the most used social media platforms.
Recruitment	We used the panel company CloudResearch to collect the data. We discuss limitations of the sample in the manuscript:<br><br>It is also important that, although the sample showed close resemblance of many core demographic variables of the U.S. population on Twitter (currently X), it was not fully representative, somewhat limiting its generalizability. In particular, women, Democrats, and Republicans were slightly overrepresented. This is discussed in the article.
Ethics oversight	The present research was approved by the Bioethics Committee of Jagiellonian University in Krakow (No 1072.6120.12.2022, January 26th, 2022). Informed consent was obtained from all participants.

Note that full information on the approval of the study protocol must also be provided in the manuscript.

Field-specific reporting

Please select the one below that is the best fit for your research. If you are not sure, read the appropriate sections before making your selection.

☐ Life sciences ☒ Behavioural & social sciences ☐ Ecological, evolutionary & environmental sciences

For a reference copy of the document with all sections, see nature.com/documents/nr-reporting-summary-flat.pdf

Behavioural & social sciences study design

All studies must disclose on these points even when the disclosure is negative.

Study description	The study is correlational and quantitative.
Research sample	A sample reflecting the population of U.S. users on Twitter (currently X) combined with data of 7.79 million engagements on the social media platform over the course of the COVID19 pandemic. The sample was close to representative. Sample limitations are discussed in the manuscript.
Sampling strategy	The data were collected via stratified sampling by an online panel company (CloudResearch) using quotas to ensure representativeness. The data structure is nested and utilizes big behavioral data, ensuring a multiple of the power required to to detect small level 2 effects in multi-level models (see https://link.springer.com/chapter/10.1007/978-3-642-72087-1_17 and https://doi.org/10.1027/1614-2241.1.3.86). Selection biases are addressed through the quota approach but cannot be fully excluded. However, as we discuss in the article, assessments of means and distributions on central variables did not suggest the presence of notable selection biases.
Data collection	The data were collected online via a survey hosted on Qualtrics and via the Twitter (currently X) Academic API.<br>In the survey, we assessed:<br>Age<br>Education<br>Gender<br>Political Orientation<br>Political Party Affiliation<br>Belief in False Information<br>Disbelief in True Information<br>Conspiracy Mentality<br>Narcissism<br>Denialism<br>Need for Chaos<br>Belief in Information Reliability<br>Importance of Verifying Information<br>Perceived Ability to Recognize Misinformation
Timing	Self-report data: August 2022 and February 2023; Social media engagement data: December 2019 to December 2021 [Data extraction took place between January 10, 2023, and March 16, 2023]; GPT Classification: April 2024.
Data exclusions	No participants were excluded.
Non-participation	3 participants withdrew their informed consent and their data were deleted.
Randomization	The study was not experimental. Main covariates are controlled for in the analyses. These are Age, Education, and Gender.

Reporting for specific materials, systems and methods

We require information from authors about some types of materials, experimental systems and methods used in many studies. Here, indicate whether each material, system or method listed is relevant to your study. If you are not sure if a list item applies to your research, read the appropriate section before selecting a response.

Materials & experimental systems

n/a	Involved in the study	
☒	☐	Antibodies
☒	☐	Eukaryotic cell lines
☒	☐	Palaeontology and archaeology
☒	☐	Animals and other organisms
☒	☐	Clinical data
☒	☐	Dual use research of concern
☒	☐	Plants

Methods

n/a	Involved in the study	
☒	☐	ChIP-seq
☒	☐	Flow cytometry
☒	☐	MRI-based neuroimaging

April 2023

Plants

Seed stocks	NA
Novel plant genotypes	NA
Authentication	NA
